# A qualitative study on redefining normality in relatives of patients with advanced cancer

**DOI:** 10.1002/cam4.7211

**Published:** 2024-05-24

**Authors:** Helen P. A. Driessen, Evi M. Bakker, Judith A. C. Rietjens, Khanh L. N. Luu, Marjolein Lugtenberg, Frederika E. Witkamp, Leonieke W. Kranenburg

**Affiliations:** ^1^ Department of Psychiatry, Section of Medical Psychology and Psychotherapy, Erasmus MC University Medical Center Rotterdam Rotterdam The Netherlands; ^2^ Erasmus MC Cancer Institute Erasmus MC, University Medical Center Rotterdam Rotterdam the Netherlands; ^3^ Department of Public Health, Erasmus MC University Medical Center Rotterdam Rotterdam The Netherlands; ^4^ Department of Design, Organization, and Strategy, Faculty of Industrial Design Engineering Delft University of Technology Delft The Netherlands; ^5^ Department of Dermatology Erasmus MC, University Medical Center Rotterdam Rotterdam The Netherlands; ^6^ Scientific Center for Care and Welfare (Tranzo), Tilburg School of Social and Behavioral Sciences Tilburg University Tilburg The Netherlands; ^7^ Research Center Innovations in Care Rotterdam University of Applied Sciences Rotterdam The Netherlands

**Keywords:** accommodation, adaptation, psychological, advanced cancer, assimilation, family, neoplasms, palliative care, qualitative research

## Abstract

**Objective:**

To obtain insight into adaptation processes of redefining normality and its influencing factors in relatives of patients with advanced cancer.

**Methods:**

An exploratory qualitative study among relatives of patients with advanced cancer was conducted. Participants were purposively recruited. Ten in‐depth individual (relative only) and 16 dyad (relative and patient together) interviews were conducted, transcribed verbatim, and analyzed by means of thematic analysis, drawing on elements of grounded theory, combining both inductive and deductive elements.

**Results:**

Two adaptation processes of (redefining) normality were identified: assimilation and accommodation. The latter was found to be the main way of adapting to new events. Assimilative coping strategies entailed “continuing to do the same activities as done before the disease,” “difficulty accepting the situation,” “avoiding to think about the disease,” and “living in the short term.” Accommodative strategies involved “arranging practical matters,” “thinking about the future,” “doing what is feasible,” “engaging in new activities,” “accepting the situation,” “seeking distraction,” “living in the short term,” and “focusing on what truly matters in life.” The interplay between the diagnosis and treatment of cancer, a deteriorating disease status, and the accompanying uncertainty about the future was of influence on the relatives' coping strategies.

**Conclusion:**

When the new situation is too divergent to assimilate, accommodation may be necessary for relatives to cope with the growing complexity of the consequences of their loved one's illness. Accommodative coping then involves accepting the changing reality and actively making the necessary adjustments to build resilience and cope with the new circumstances.

## Graphical abstract

Redefining normality fluctuates through processes of assimilation and accommodation. Diagnosis and treatment, deteriorating health, and uncertainty about the future influence the relatives' coping in redefining normality. Relatives predominantly opt for accommodation over assimilation in coping with advanced cancer.

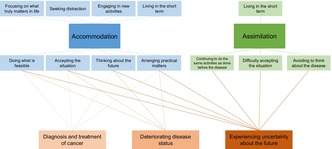

## 1 INTRODUCTION

Relatives of patients with advanced cancer increasingly have to take on the responsibility of caring for the patient.^1^ They often spend 18–33 h a week on caring for their loved ones.^2^, ^3^ This impacts familial, social, work, and financial related aspects of their life,^4^, ^5^, ^6^ and they struggle to find a balance in the ever‐changing conditions of daily life.^7^ Patients and relatives often try to adapt to the new situation by maintaining or striving for *normality* as much as possible.^7^, ^8^, ^9^

In the context of patients and relatives in the advanced stage of cancer, *normality* on the one hand encompasses trying to continue to live life as prior to the diagnosis as much as possible.^7^, ^8^, ^9^, ^10^, ^11^, ^12^, ^13^ On the other hand, normality relates to seeking reliable patterns of everyday life while adapting to unaccustomed and unprecedented life situations.^9^, ^14^ In the phase after the diagnosis of advanced cancer, patients and relatives often experience uncertainty and fear, because they are confronted with limited time and they perceive “disruption,”^15^, ^16^ which refers to “being in the midst of living and dying.”^16^ To counteract these feelings, relatives redefine their reality and recreate a sense of pattern in the uncertainty and chaos, to come to terms with their new situation.^7^ This *abstract* process is known as “redefining normality.” Relatives live through the same process of redefining normality as patients.^9^ For people diagnosed with cancer, striving for normality distracts them from the preeminent perception of being a patient. Behaving in a fashion that allows them to remain “normal” is essential for maintaining integrity. Because “by ‘carrying on as normal’ and focusing on the present, people can maintain a sense of purpose and hope for themselves and close family members.”^17^ Or, as van Roij and colleagues state: “being able to do the same things also gave them a feeling of control, satisfaction, meaning, and social embeddedness.”^8^

Redefining normality can help patients with advanced cancer and their relatives cope with fear and uncertainty about the future.^9^, ^13^ It can be seen as a strategy to deal with restrictions on making plans for the future.^9^ Furthermore, thinking of death provokes painful feelings. A daily routine provides a distraction from the disease and has a relieving effect.^9^ Within striving for normality in the palliative context, patients and their relatives also try to appreciate life through “living in the moment.”^17^, ^18^, ^19^ Living from day to day helps to focus on the actual moment and to not become overpowered by speculations about the future. Relatives find it hard to “achieve a balance […] to deal with their own lives and with their everyday life with the ill person, balancing between the old and the new life situations.”^20^ By planning in the short term, relatives can maintain daily life, but constantly with some uncertainty, as everyday planning depends on the patient's health status.^20^, ^21^, ^22^ Normality through routine activities is seen as an interconnected effort of relatives to cope with their own emotions and those of the patient.^9^ It gives relatives the ability to manage the demands of their caregiving role, with a degree of certainty, a sense of confidence, and control.^14^

Previous qualitative research with respect to dealing with advanced cancer was more focusing on coping. Coping with advanced cancer is a complex and dynamic process and seems to arise from minor changes in day‐to‐day behavior and attitudes.^18^ Although specific goals and preferences will differ between relatives, their desire to maintain normality is common.^8^, ^9^, ^10^, ^11^, ^12^, ^13^, ^14^, ^18^ However, research into what relatives *actually* mean by this, and how they shape this, is lacking. In this study, we aim to gain insight into adaptation processes of redefining normality and its influencing factors in relatives of patients with advanced cancer.

## 2 METHODS

### 2.1 Study design and population

The present explorative study is nested in a qualitative study in which in‐depth interviews were conducted with patients with advanced cancer and their relatives, to gain understanding of their challenges in self‐management.^19^ Both studies adhere to the COREQ (COnsolidated criteria for REporting Qualitative research) Checklist for qualitative research.^20^ Patients were eligible when they were 18 years or older, were diagnosed with any type of advanced cancer, and were able to sign the informed consent form. Advanced cancer was defined as cancer that could no longer be cured.^21^ To be eligible for inclusion, relatives had to be 18 years or older, take care of a patient with advanced cancer, and were able to sign the informed consent form. Relatives could be, for example, partners, children, other family members, or friends. In the original qualitative study,^19^ we were interested in the perspectives of the persons with cancer and their relatives To be attentive to the preferences of the participants, we allowed individual interviews and dyadic interviews. Patients or relatives could participate in the study irrespective of the participation of the other. Participants were purposively recruited to represent different educational levels and cultures.

General practitioners, oncologists, and nurses from one general hospital, one academic hospital, and two hospices asked eligible patients whether they wanted to participate in the study. Potential participants were contacted by phone to explain the study and were asked whether they had a relative who potentially wanted to participate. Calls for partaking were also circling via websites of a patient organization, and several research and social media platforms. Additional information was sent by postal mail. The informed consent form was signed prior to the interview. Individual interviews were conducted with patients and relatives, as well as dyad interviews involving both patients and their relatives. The interviews were held at a location of the participants' choosing and most interviews were conducted at their homes. Travel expenses were reimbursed. Interviews were conducted until no new information with relevance to the research question emerged and saturation was achieved. The interviewers (HN, RS, KL, MA, and JR) had backgrounds in anthropology, psychology, nursing (KL and MA), and health sciences, respectively. For the individual interviews, it took on average 45 min (range 20–70 min) until saturation was reached. For the dyad interviews, it took on average 50 min (range 25–70 min) until saturation was reached. The Medical Ethical Committee of the Erasmus Medical Center reviewed and granted exemption from ethical review for this study, according to the Dutch Medical Research Involving Human Subjects Act (WMO) (MEC‐2018‐1368).

### 2.2 Data collection

To produce an in‐depth understanding of the challenges patients and relatives experience in self‐managing the disease, an interview guide was developed prior to starting the interviews (Box 1).^19^ The guide was partly based on a systematic review on self‐management of patients with advanced cancer.^22^ Interviews were conducted between September 2018 and July 2019. The interviews were audiotaped and transcribed verbatim. Interviewers also took field notes. Additional information on the research team and reflexivity of the original study is reported elsewhere.^19^ After the interviews, participants completed a questionnaire on demographic characteristics (e.g., type of cancer, year of diagnosis, and current treatment).

BOX 1 **Interview guide used in conducting the interviews**

1. Can you tell me something about yourself and your situation?
2. What consequences does your relative's disease have on your life?
3. What helps you to deal with the consequences you described?
4. Which of the consequences bothers you most?
5. What could help you to better manage your relative's disease and its consequences?

### 2.3 Data analysis

For the present study, we performed a thematic analysis, drawing on elements of grounded theory, combining both inductive and deductive elements.^23^ We used open, axial, and selective coding,^24^ supported by NVivo 14. We used data from 10 individual interviews with relatives of patients with advanced cancer, as well as 16 dyad interviews involving both patients with advanced cancer and their relatives. With respect to the dyad interviews, we only analyzed transcriptions involving the relatives. EB (medical student) and KL (nurse and health scientist) both first read five transcripts of individual interviews and identified initial codes (open codes), which were discussed with members of the research team, with backgrounds in nursing, psychology, oncology, social sciences, epidemiology, and health sciences.

In this initial phase of coding, the data were found to fit a theory on coping, namely that of *assimilation* and *accommodation*.^25^ Initially proposed according to Piaget's theory, assimilation and accommodation are two complementary processes of adaptation that involve the utilization and alteration of perceptions.^25^ The theory of assimilation and accommodation as ways of coping, as proposed by Brandstädter,^26^ denotes different modes of achieving congruence between actually perceived and desired circumstances. The theory of Brändstadter was subsequently utilized for the development of our own model (see Box 2).

BOX 2 **The theory of assimilation and accommodation, as proposed by Brandstädter**

The theory of Brandstädter refers to means of attaining congruence, or neutralizing, or coping with differences, between the real and desired situation, mostly in the context of aging. Assimilative coping refers to tenacious goal pursuit and accommodative coping implies flexible goal adjustment. Subjective life‐quality over the life course depends not only on efficient goal pursuit, but also on the readiness to adjust goals and ambitions to the feasible range. Assimilative processes involve efforts to achieve and maintain desired courses of personal development, as well as intentional activities to prevent, or compensate for, functional losses and impairments. Assimilative persistence helps to stabilize goal pursuit against obstacles and distractive influences. On the other hand, facets of accommodation include downgrading of, and disengagement from, blocked goals and reorientation, rescaling of ambitions and self‐evaluative standards, positive reappraisal of the status quo, and finding positive meaning in losses and aversive life changes which appear irreversible. The key characteristic of this adaptive mode is the flexible adjustment of previously adopted goals and ambitions to situations that cannot be changed or controlled. Accommodative activities help to maintain well‐being and self‐esteem when goals drift beyond the feasible range and losses become irreversible.

Next, we read the remaining individual and dyad interviews, identified more open codes, and composed an initial coding tree. During the phase of the constant comparative method (axial coding), we deductively coded and contextualized the data within this existing theoretical framework. EB and KL analyzed three individual and two dyad interviews. They composed a new coding tree, which was then tested by EB, KL, HD, and YF on another five transcripts. Members of the research team (EB, HD, YF, LK, FW, ML, and JR) met several times to discuss and refine the coded transcripts, themes and subthemes, and each theme in relation to the other themes. The coding tree was adjusted accordingly. Subsequently, all transcripts were revisited with the final coding tree by EB, HD, and YF. After 26 interviews, we identified no emergence of new (sub) themes, made little or no changes to the codes and we reached thematic saturation.^23^ We then organized the findings according to the main themes (selective coding).

The qualitative rigor of our study was enhanced by encouraging confirmability and reflexivity in regular meetings of the research team and discussing potential biases related to the study topic. Additionally, the interviewers kept detailed notes that encouraged them to reflect not only on the interview content but also on their own emotions during the interviews. Analytical rigor was increased by the use of dual coding and the active exploration of the researchers' different views during the analysis. Credibility was enhanced through investigator triangulation, in which a diverse team of researchers took part in the coding and interpretation of the data.

## 3 RESULTS

Fourteen (54%) relatives were female (Table 1), and their age ranged from 22 to 80 years. In most cases, the relative included in our study was the patient's spouse (85%). Twenty‐five participants had the Dutch nationality, and one participant had the Colombian nationality. The 10 participants that were interviewed individually were recruited via multiple ways: four via the general hospital, three via an academic hospital, two via social media, and one through the website of a patient association. The 16 dyads interviewed were recruited as followed: ten times in the general hospital, four times in an academic hospital, once in the hospice, and once through the website of a patient association. Breast cancer was the type of cancer that most patients were diagnosed with (42%).

**TABLE 1:** The characteristics of enrolled relatives.

Sample characteristic	Sample description	
	Individual (*n* = 10)	Dyad (*n* = 16)
Gender, *n* (%)		
Male	6 (60.0%)	6 (37.5%)
Female	4 (40.0%)	10 (62.5%)
Age (years), median (range)	57 (31–68)	65 (22–80)
Nationality, *n* (%)		
Dutch	9 (90.0%)	16 (100.0%)
Other^a^	1 (10.0%)	0 (0%)
Nature of relationship, *n* (%)		
Spouse	7 (70.0%)	15 (93.8%)
Son or daughter	1 (10.0%)	1 (6.2%)
Brother or sister	2 (20.0%)	0 (0.0%)
Education level, *n* (%)		
Lower secondary education or less	1 (11.1%)	8 (50.0%)
Upper secondary education	3 (33.3%)	5 (31.3%)
Higher vocational or academic	5 (55.6%)	3 (18.7%)
Missing	1	0
Religious, *n* (%)		
Yes	6 (66.7%)	8 (50.0%)
No	3 (33.3%)	8 (50.0%)
Missing	1	0
Type of cancer, *n* (%)		
Colon	1 (10.0%)	3 (18.8%)
Lung	2 (20.0%)	2 (12.5%)
Breast	5 (50.0%)	6 (37.5%)
Prostate	0 (0.0%)	2 (12.5%)
Other^b^	2 (20.0%)	3 (18.8%)
Time since diagnosis (years), median (range)	3 (<1–16)	4 (<1–12)
Time since diagnosis (years), *n* (%)		
<1	3 (30.0%)	2 (12.5%)
1–5	5 (50.0%)	9 (56.3%)
6–10	1 (10.0%)	4 (25.0%)
>10	1 (10.0%)	1 (6.3%)
Current treatment, *n* (%)		
None/wait and see	0 (0.0%)	4 (25.0%)
Palliative care	1 (10.0%)	1 (6.3%)
Chemotherapy	4 (40.0%)	7 (43.8%)
Hormonal therapy	1 (10.0%)	2 (12.5%)
Targeted therapy	1 (10.0%)	0 (0.0%)
Immunotherapy	0 (0.0%)	1 (6.3%)
Surgery	1 (10.0%)	0 (0.0%)
Combination therapy	2 (20.0%)	1 (6.3%)

^a^ One relative had a Colombian background.

^b^ Other cancer types included peritoneal, penile, cervical, liver, and esophagus.

Further, the results are described in two parts: the first part describes normality as an adaptive strategy of relatives of patients with advanced cancer and the second part describes the factors that are of influence on the relatives' coping.

### 3.1 Normality

**FIGURE 1:**
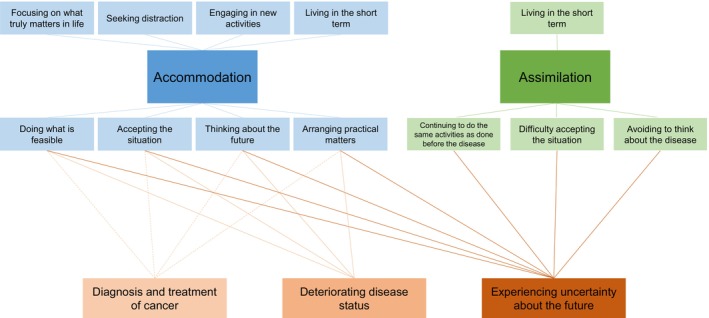
Thematic map showing two main themes and its subthemes of the adaptation processes of redefining normality and its influencing factors.The two main themes of assimilation and accommodation are situated in the second row. The factors that influenced the relatives' coping strategies are situated in the fourth row. Subthemes o assimilation and accommodation that were not related to the influencing factors are situated on the first row. Subthemes of assimilation and accommodation that were related to the influencing factors are situated on the third row.

Participants utilized both assimilative and accommodative coping strategies, which were considered the main themes (Figure 1). In general, we identified accommodation as the dominant coping strategy. Moreover, both assimilation and accommodation are divided in multiple subthemes, which are positioned above and below the main themes in Figure 1.

#### 3.1.1 Assimilation

The most prominent subtheme of assimilation we identified was “continuing to do the same activities as done before the disease.” Examples of this included continuing to pursue work or personal hobbies or participating in family activities as usual, without adapting to the new situation. Further, participants clearly indicated that they experienced “difficulty to accept the situation, and they avoided to think about the disease.” A somewhat less occurring subtheme we identified was “living in the short term.”

#### 3.1.2 Accommodation

The most prominent subtheme of accommodation we identified was “arranging practical matters.” For instance, participants mentioned arranging a will, taking care of the patient and taking over household tasks, and making adjustments to their homes to enable the patient to continue to live at home for as long as possible. Next to this, they mentioned preparing for conversations with healthcare providers to gather more information on, for example, alternative treatment options. Another frequently occurring subtheme we identified was “thinking about the future,” which included contemplating that the patients' life might be over soon, planning a final vacation together with the family, and thinking of how to resume life in general once the patient would pass away. Additionally, “doing what is feasible” was identified frequently, involving adjustments in household tasks, hobbies, and vacations. Another subtheme we identified was “accepting the situation.” To a somewhat lesser degree, we identified the subtheme of “seeking distraction,” which involved performing work‐related tasks in a modified manner, engaging in personal hobbies, or engaging in family activities. We also identified the subtheme “engaging in new activities,” in which the relatives took on activities that they did not pursue before the diagnosis, such as new hobbies or jobs. Lastly, “living in the short term,” and “focusing on what truly matters in life” were identified subthemes, respectively.

#### 3.1.3 Interrelatedness of assimilation and accommodation

Relatives use both accommodative and assimilative coping modes in striving for normality. In some instances, the interrelatedness of both concepts became apparent within one text fragment, for example, “accepting the situation” and “difficulty accepting the situation”: “At times, which I can genuinely count on one hand, I've had moments where I think, you know… darn it, usually I just accept it all, but there are those moments when you think, I wish we were just normal, not ill, and we could do everything again as husband and wife, you know. […] You just get completely fed up, even though that feeling never lasts long.”(R031, patients' spouse, less than a year since diagnosis, female, age unknown).

Further, we identified the concept of “living in the short term” as a coping strategy for both assimilation and accommodation, although this theme was more present as an accommodative strategy. Participants adopting an assimilative coping mode indicated that they “lived in the short term” simultaneously with “avoiding to think about the disease.” Participants with an accommodative coping mode on the other hand indicated that they “lived in the short term” as they simultaneously “accepted the situation as it was” and “focused on what really matters in life.”

The same principle holds for the concept of engaging in activities such as hobbies and work. This theme was identified more frequently as an assimilative coping mode. The concept of assimilation involves “continuing to do the same activities as done before the disease,” as a form of denial of, or refusal to recognize, the new circumstances. Conversely, the concept of an accommodative coping mode was characterized by a way of “seeking distraction” from something they acknowledge to be present in their lives.

Further, participants recognized striving for normality as a mean to regulate their emotions. Assimilative coping modes served to regulate emotions by ignoring or suppressing them, believing that “it is not appropriate to feel sad or worried at this stage of the disease” and “avoiding to think about the disease”: “You don't think about it too much, and you make sure not to think about what's coming. Because, well, firstly, that doesn't really serve much purpose in changing the situation, and secondly, it just makes you sad anyway, so that doesn't really help much in the end.” (R011, patients' daughter, 1 year since diagnosis, female, 45 years). An accommodative coping mode functioned as such by “enjoying positive aspects of their current life” and “maintaining control over their feelings and the situation.” As a result, they approached the situation constructively: They emphasized the importance of “living in the short term,” “focusing on what truly matters in life,” and shifting their focus to “doing what is feasible”: “[…] by living day by day and not looking too far ahead. And that's what I tell others: things are going well now, but I don't know about next week, so we're just enjoying how things are going at the moment.”(R011, patients' daughter, 1 year since diagnosis, female, 45 years). To illustrate the intrapersonal contrast, we have referenced two quotes from the same relative: both coping modes can exist within one individual in striving for normality.

### 3.2 Factors that influence how relatives strive for normality

We identified three important factors that influenced the relatives' coping strategies in striving for normality: (1) the diagnosis and treatment of cancer, (2) a deteriorating disease status, and (3) experiencing uncertainty about the future. Table 2 presents quotes of relatives, categorized per subtheme and per influencing factor, in which the influence of these factors on the strategies to strive for normality became apparent. These influencing factors are presented in Figure 1 and are positioned at the bottom of the figure. Positioned above the main themes, are subthemes of assimilation and accommodation for which no clear relationship between the influencing factors was found. Positioned below the main themes, are the subthemes for which we identified a relationship between the influencing factors and the relatives' coping strategies.

**TABLE 2:** An overview of factors that influenced the relatives' strategies to strive for normality and illustrative quotes, categorized per (sub) theme.

Factors that influence normality	Quotes
Diagnosis and treatment of cancer	Assimilation ‐
	Accommodation *Doing what is feasible* R: “[…] And the doctor said, ‘Well, we'll have to perform surgery’ and then asked ‘do you want to do it now?’, because we were supposed to go on vacation. Well, in the end, we did go on vacation, just for a week… a week together with our kids… And he asked, ‘Do you want to do it after your vacation or during your vacation?’ Well, it ended up being during the vacation. So, we were away for about four days, and then we had the surgery during that vacation.” (R018, patients' spouse, 9 years since diagnosis, male, 58 years) *Accepting* R: “I mean, we heard yesterday that this is the final treatment. If it goes wrong, it's probably fatal. And also acceptance. Acceptance, we know it's like that. The disease, you shouldn't speak in terms of winning and losing, we're not going to… it's going to catch up to us, and my wife is going to pass away from it. We've known that for a very long time, actually since 2013.” (R005, patients' spouse, 5 years since diagnosis, male, 31 years) *Arranging practical matters* R:“She was admitted to the hospital. and well, I'm the primary contact person. It started with her admission […], then she went home, but of course, she had to undergo surgery to have everything removed. And the radiation therapy and chemotherapy sessions, all of it was part of the first year of treatment. So, I would take her there, pick her up, and take care of meals. She had to inject herself with shots for a while, so I would go to her every evening to administer the injection. Yes, it was very intensive.” (R012, patients' sister, 3 years since diagnosis, female, 48 years)
Deteriorating disease status	Assimilation ‐ Accommodation *Doing what is feasible* R:“You have your own routines and responsibilities throughout the day: your work, your free time. And now that we are in a very advanced stage of the disease, the disease is becoming increasingly dominant; it dominates my agenda.” Interviewer: Also in your life. R:“Yes, two weeks ago, for the first time, I sort of set aside my work. I cancelled everything […] and this has been the first time in the whole disease trajectory because I have a strong work ethic. But then you realize, ok, now the disease is dominant in life, and I have to align my work with it.” (R005, patients' spouse, 5 years since diagnosis, male, 31 years) *Accepting* Interviewer: “How did you react to that phone call, to the news about the bad prognosis?” R:“Well, you just have to accept it and process it together.” (R016, patients' spouse, 9 years since diagnosis, male, 71 years) *Thinking about the future* R:“[…] most challenging moment for me was when we received the news about the metastasis; I was devastated by that. Yes, your whole world just crumbles. That's when you start contemplating about the future as well.” (R007, patients' spouse, 1 years since diagnosis, male, 29 years) *Arranging practical matters* R:“I've seen the scan myself. Then you start thinking: how much time does he have left? The lung specialist said about three months. […] Well, then you quickly book a week of vacation and start taking care of financial matters.” (R029, patients' spouse, 4 years since diagnosis, female, 78 years)
Experiencing uncertainty about the future	Assimilation *Avoiding to think about the disease* R: “Next week, she's getting her third round of chemotherapy, and that's incredibly nerve‐wracking… will it prove effective again? And then, you do find yourself sitting at that desk with trembling knees, of course.” Interviewer: Yes, I believe that. R: “Because it's now the third time she's undergoing chemotherapy and it's getting more and more intense and nerve‐wracking… Because if this doesn't work, then what? And I have to admit that I immediately erase that thought from my mind, because that's something I can't handle at the moment, you know. […] I think, okay, I need to stop my thoughts from running, and refrain from dwelling on it, and focus on living in the short term.” (R012, patients' sister, 3 years since diagnosis, female, 48 years)
	*Difficulty accepting the situation* R: “[…] what has been playing on my mind lately, as strange as it may sound, is the fact that it's taking longer and longer, and it's getting heavier and heavier. Just because it takes so long.” Interviewer: “Yes, I can imagine that very well. Yes, it gets heavier for you too.” R: “*Yes*.” Interviewer: “Yes, of course. Is it heavier primarily to live with that thought, or is it also heavier in terms of the caregiving tasks?” R: “No […] the caregiving tasks are peanuts, that's not a problem at all. Even if it gets worse later, that's not an issue for me at all.” Interviewer: “You can organize that.” R: “Yes, we'll manage that; that's not the point. But it's just accepting, realizing that she won't be here anymore…” (R004, patients' spouse, 16 years since diagnosis, male, 68 years) *Continuing to do the same activities you did before the disease* Interviewer: “And how is it for you to live with that knowledge that every day, as you just mentioned, you're prepared for the worst?” R: “Yes, that does come up every day… I just see it as a kind of survival: I just try to let life go on and not letting it take the lead, even though it's incredibly important because you deal with it every day… But we went skiing, and he just went along as well.” (R020, patients' spouse, 12 years since diagnosis, female, 60 years)
	Accommodation *Doing what is feasible* Patient: “One of our children lives in Sweden, and we had plans to visit them last summer, but we had to cancel all of that. Now, as I start feeling a bit better, I'll gain more strength, and then maybe we can plan a short trip or a few days away.” R: “Yes, and for now, we'll stay close by, at least in the Netherlands.” You can't book a trip to Spain or anywhere else right now […]. In December, we want to go back to Sweden… Patient: “Yes, give it another try…” R: “But then you think, maybe something will come up again, and we'll have to cancel it again…” Patient: “Yes, disappointing the children once more.” R: “Yes, it's really difficult to make plans.” (R013, patients' spouse, less than a year since diagnosis, male, 62 years) *Living in the short term* R: “[…] If I had to mention something, it would be the uncertainty about the future that keeps coming up, not knowing how things will go. Is it going to stay this good? […] People always ask, ‘How is your mother doing?’ I say, ‘She's doing really well now, but I don't know about tomorrow or next week’.” Interviewer: Have you found a way to cope with that uncertainty? R: “Yes, by living day by day and not looking too far ahead. […] We just enjoy how things are going at the moment.” (R011, patients' daughter, 1 year since diagnosis, female, 45 years) *Accepting the situation* R: “But yes, statistically speaking, the chance of her living another 5 years is only 2%, so you have to consider… I always have in the back of my mind that we might hear one day, ‘It has grown again, and there's nothing more we can do’.” Interviewer: How do you deal with that thought? R: “Accept it, there is no other way. Again, I'm not a wizard, and I'm not a doctor. If I could perform magic, she wouldn't be sick. If I could perform magic, then…” (R010, patients' spouse, 4 years since diagnosis, male, 62 years) *Thinking about the future* R: “And of course, and this is quite essential, if there is indeed a partner, me in this case, you naturally go through a grieving process in advance. That's also part of it. And that dark cloud, that sword of Damocles hanging over your head, colors each day anew. […] And it doesn't go away, those worries. The relative carefreeness I once had […], it's gone and it will never come back. When you've been together for 45 years, […] it's like an impending amputation. You're still a team, a unity, that's what we always said, and it seems like that's coming to an end, and it's not a pleasant prospect.” (R031, patients' spouse, less than a year since diagnosis, female, age unknown) *Arranging practical matters* R: “[…] We discussed the custody of our children, we talked about the funeral, that's all taken care of. We've thought through everything about how it might… how the end could be.” (R005, patients' spouse, 5 years since diagnosis, male, 31 years).

#### 3.2.1 Diagnosis and treatment of cancer

The diagnosis and treatment of the disease influenced participants displaying accommodative strategies in striving for normality. This factor did not seem to affect relatives who used assimilative strategies.

#### 3.2.2 Deteriorating disease status

A deteriorating disease status refers to identifiable moments of deterioration of the patients' health. In case of assimilation, a deterioration of the patient's health did not directly lead to behavior change. Naturally, there is a limit to this approach as, at a certain point, denial becomes untenable. In case of accommodation, being aware of a disease status that undeniably worsens, prompted participants to actively address the new reality and adapt to the altered circumstances.

#### 3.2.3 Experiencing uncertainty about the future

Experiencing uncertainty about the future influenced both assimilative and accommodative strategies to strive for normality.

## 4 DISCUSSION

In our study, we observed that, in one way or another, all relatives strive for a sense of *normality* as a way to cope with the situation in which their loved one is facing incurable cancer. However, *how* people strive for normality is not uniform and static within a person, but rather dynamic, depending on the context and specific situation. We found that *striving for normality* among relatives of patients with advanced cancer is not experienced or understood in a single way, but rather through two different modes: assimilation and accommodation. Moreover, both assimilative and accommodative coping modes may occur within one person, especially when new information and experiences challenge one's existing norms and notions of normality, but still allow for some recognition and integration.

It can be hypothesized that if the deteriorating disease status and the accompanying experienced uncertainty about the future are not prominently present and thus not all‐encompassing in one's life, a short‐term “way out” to assimilation remains. Assimilation often becomes less tenable as the situation undeniably worsens, making the situation too divergent to assimilate, and thus, relatives tend to, or maybe even “have to” accommodate. The reality of a deteriorating disease status then prompts relatives to actively address the new reality, for instance by “arranging practical matters.” These findings match the general conception of assimilation and accommodation as developmental processes. As long as circumstances do not “force” one to, it is easier and in a sense more economical (in terms of saving energy) to assimilate.^27^ This, however, has the risk of becoming disconnected from reality and running into a somewhat isolated and eventually untenable position. For someone who thus far has upheld assimilation, the “sudden” death of a relative may then impair further processing of the event, and even lead to disorders of posttraumatic stress disorder.^28^ As such, parallels can be drawn with multiple conceptions of assimilation and accommodation. In their review of assimilation and accommodation, Hanfstingl et al. have identified research areas in which assimilation and accommodation are applied.^29^ The areas that seem most relevant for explaining our findings are, respectively, those of “personality and identity” and “coping.” We will address both areas below, in relation to our findings.

First, from the research area of personality and identity, especially the works of Wortmann and Park and Proulx and Inzlicht seem of relevance.^30^, ^31^ These authors focus on the importance of assimilative and accommodative processes for meaning‐making when dealing with loss. This exactly has been the topic of our study, and our findings corroborate their specific conception of assimilation and accommodation. According to their theory, assimilation refers to processes wherein meaning‐making changes situational appraised meaning to be more consistent with already existing global meaning, and accommodation is associated with changing pre‐existing global beliefs or goals. More specifically, Proulx and Inzlicht go into common compensation behaviors that follow from violations of our committed understanding, as is the case as one is confronted with the situation that one's relative is dying of cancer before the “expected” end of life. According to their model, all “meaning violations” may bottleneck at neurocognitive and psychophysiological systems that detect and react to the experience of inconsistency. This in turn motivates compensatory behaviors. Such compensatory behavior includes, among others, both retaining a sense of the familiar (assimilation) and restoring a sense of familiarity (accommodation). Both behaviors match our findings. For instance, retaining a sense of the familiar was found in the subtheme of “Continuing to do the same activities as done before the disease” and resetting a sense of familiarity clearly comes across in the subtheme “Doing what is feasible” (Figure 1). With regard to assimilation and accommodation as applied to the field of coping research, it is important to realize that these dimensions are not opposed to each other, but each form the positive side of the coin of helplessness (in case of assimilation) and rigid perseverance (in case of accommodation).^32^ This conception of terms is helpful in understanding our findings, in the sense that there is not one (single) good tendency to cope, but that instead both tendencies (assimilation and accommodation) can be helpful in adjusting to the appalling conditions in which a relative is close to dying of cancer. This conception is also in line with the concept of “Double Awareness” as described by Burgers et al. in their study on adolescents and young adults (AYAs) with uncertain or poor cancer prognosis.^33^ While coping with their cancer, AYAs walk on two paths at the same time: “one path focusing on engagement in life and one path focusing on the reality of premature death.” This is influenced by treatment regimens and disease progression. By “living according to the metaphor carpe diem and being consciously alive,” AYAs strive to find a new balance in life and can reach double awareness.

In addition, it may also be of worth to view coping not from an individual, but from a couple's perspective, often described as dyadic coping. Dyadic coping refers to the process wherein stress and coping are viewed as interpersonal processes.^34^, ^35^ Following this, a stressful event always concerns both partners. Applied to cancer, one can say that although only one partner is struck by the disease and its consequence, the other partner is too in the sense that one is worried about (losing) their partner, and has to arrange all kinds of practicalities. In dyadic coping, both partners are mutually involved in the stress coping process, continuously influencing each other, with the aim of reducing distress for both partners (“members of the dyad”) and to preserve or enhance the relationship functioning.^34^ As explained in the systemic‐transactional model, the dyadic coping process then is an interplay between (non)‐verbal stress signals, the interpretation of and response to these signals by the other partner, and joint coping efforts.^34^ In addition, the developmental‐contextual model of couples coping with chronic illness underlines that dyadic coping may differ over time and may change for instance as the disease develops.^36^ This is in line with our findings, where we found that disease progression may influence how partners cope.

In addition to assimilation and accommodation as primary coping modes or tendencies, we also identified factors influencing their application. These factors are the diagnosis and treatment of cancer, a deteriorating disease status, and experiencing uncertainty about the future. In this respect, especially the factor “deteriorating disease status” highlights the temporal or developmental dimension of adaptation. Duggleby et al. recognizes “crucial events” as influencing coping, or to put it even stronger, as moments in time when someone, in as far as one does not already do so, is elicited to embrace a more accommodative strategy.^7^ The factor “experiencing uncertainty about the future” may be most notifiable when it comes to the clinical implications of our findings. Roughly said, as long as people can do something, or literally “have a hold,” for instance by arranging practical matters, adaptation is “easy” to mold. It may become extra tough when things become abstract and thus become something someone “can't do anything with.” The abstract aspect may make it easier for someone just to ignore, as abstract things “do not take place anyway.” When at moments, such conception for sure may be helpful, complete denial is difficult to maintain. Death, however, as the ultimate abstract and unknown, can be quite impressive and it is only human to sidestep this. Nevertheless, this does not mean that professionals need to avoid the issue as well. It would make sense to initiate addressing this topic as a healthcare provider, of course not without attuning to the person in question.

The results of our research are valuable as it contributes to a better understanding of the concept of normality within this specific context. A comprehensive understanding of this concept is essential for healthcare providers to understand the coping mechanisms employed by relatives, and to meet their needs as they cope with the disease and its consequences. Thus, in practical terms, our research underscores the importance of adopting a proactive approach in communicating with relatives, taking into account the dynamic nature of striving for normality in the face of the disease. This communication serves to establish a clearer understanding of the coping modes employed, facilitating the development of a more personalized and effective support system. For instance, in cases where relatives express assimilative coping modes, there may be a lower readiness for Advanced Care Planning, potentially leading to an increased risk of end‐of‐life crises. Therefore, healthcare providers are advised to be extra vigilant during the terminal phase of care, recognizing the potential challenges and adapt their approach accordingly. The integration of these insights into clinical practice holds the potential to significantly enhance the quality of care and support provided by healthcare providers to relatives coping with the profound effects of the disease. The help of, or referral to, spiritual care professionals may also be appropriate in this context.

### 4.1 Strengths and limitations

To our knowledge, this is one of the first studies that addressed adaptation processes of redefining normality and its influencing factors in a population of relatives of patients with advanced cancer. Although the interview guide was composed to gain an in‐depth understanding of the challenges relatives experience in self‐managing the patient's disease, it allowed for new topics that were spontaneously raised. The current study has a cross‐sectional design, thereby not allowing for making comparisons over time. Future studies could use longitudinal designs, especially to gain more insight into whether accommodative tendencies indeed become more important in relatives of patients with cancer facing end of life. Furthermore, although a relatively large group of participants from a variety of backgrounds was purposively included, it turned out to be difficult to enroll persons from varying cultural/ethnic backgrounds. This in itself is an interesting (though not unique) research finding, which draws attention to the need to find strategies to ensure a more diverse inclusion in studies like ours. Further, it is important to note that we did not explore age dependency of the relatives' adaptation processes. On one hand, as one ages, it can be hypothesized that one may develop greater flexibility due to the numerous adjustments one has had to make throughout ones lives. On the other hand, it can also become more challenging to adapt, as older individuals may potentially become stuck in old habits and routines. Notably, the study conducted by Walshe et al. indicated that the relatives' coping mechanisms did not appear directly related to disease type, disease trajectory, age, gender, or demographic factors.^18^ Last, it is important to acknowledge that throughout the dyad interviews, the relatives may have been influenced by the patient's presence. On one hand, they may have been reserved, perhaps out of a desire not to cause distress to the patient. On the other hand, in the presence of someone they trusted, they may have exhibited greater openness.

## 5 CONCLUSION

The findings of our study address a gap in the literature on the perceptions and actions of normality of relatives of patients with advanced cancer. We identified assimilative and accommodative tendencies, which seem to be influenced by the diagnosis and treatment of cancer, a deteriorating disease status, and the experienced uncertainty about the future.

In general, relatives tend to accommodate to the new circumstances within the context of advanced cancer instead of attempting to assimilate. Assimilation often becomes less tenable as the situation undeniably worsens, making the situation too divergent to assimilate. The interplay between the deteriorating disease status and the accompanying uncertainty about the future influences the relatives' coping: Accommodation seems essential to cope with the growing complexity of the disease's consequences in terms of care, support, and emotional engagement. Accommodative coping then involves accepting the changing reality and actively making the necessary adjustments to build resilience and cope with the new circumstances.

## AUTHOR CONTRIBUTIONS

**Helen P. A. Driessen:** Formal analysis (lead); investigation (lead); methodology (lead); project administration (lead); software (lead); visualization (lead); writing – original draft (lead). **Evi M. Bakker:** Conceptualization (equal); data curation (equal); formal analysis (lead); investigation (lead); methodology (lead); software (equal); visualization (lead); writing – original draft (lead). **Judith A. C. Rietjens:** Conceptualization (lead); data curation (lead); funding acquisition (lead); investigation (supporting); methodology (equal); validation (equal); writing – review and editing (equal). **Khanh L. N. Luu:** Conceptualization (equal); data curation (lead); investigation (equal); writing – review and editing (equal). **Marjolein Lugtenberg:** Conceptualization (equal); investigation (equal); methodology (equal); validation (equal); writing – review and editing (equal). **Frederika E. Witkamp:** Conceptualization (equal); investigation (equal); methodology (equal); validation (equal); writing – review and editing (equal). **Leonieke W. Kranenburg:** Conceptualization (equal); formal analysis (lead); investigation (equal); methodology (equal); supervision (lead); validation (equal); writing – review and editing (lead).

## FUNDING INFORMATION

91717386/Dutch Research Council (NWO), Innovational Research Incentives Scheme Vidi.

## CONFLICT OF INTEREST STATEMENT

The authors declare no conflicts of interest.

## ETHICS STATEMENT

The Medical Ethical Committee of the Erasmus Medical Center reviewed and granted exemption from ethical review for this study, according to the Dutch Medical Research Involving Human Subjects Act (WMO) (MEC‐2018‐1368).

## HUMAN AND ANIMAL RIGHTS AND INFORMED CONSENT

Informed consent is obtained from all study participants. This article does not contain any studies animal subjects performed by any of the authors.

## Data Availability

The data that support the findings of this study are available on request from the corresponding author. The data are not publicly available due to privacy or ethical restrictions.
