# Correction to A qualitative study on redefining normality in relatives of patients with advanced cancer

**DOI:** 10.1002/cam4.70151

**Published:** 2024-08-28

Driessen HPA, Bakker EM, Rietjens JAC, Luu KLN, Lugtenberg M, Witkamp FE, Kranenburg LW. A qualitative study on redefining normality in relatives of patients with advanced cancer. *Cancer Med*. 2024 May;13(10):e7211. doi: 10.1002/cam4.7211. PMID: 38785201; PMCID: PMC11117454.

An error occurred in the publication process, resulting in the incorrect listing of the authors' affiliations.

The correct affiliations are listed below:

**Helen P. A. Driessen**

‐ Department of Psychiatry, Section of Medical Psychology and Psychotherapy, Erasmus MC, University Medical Center Rotterdam, Rotterdam, The Netherlands
‐ Erasmus MC Cancer Institute, Erasmus MC, University Medical Center Rotterdam, Rotterdam, The Netherlands

**Evi M. Bakker**

‐ Department of Public Health, Erasmus MC, University Medical Center Rotterdam, Rotterdam, The Netherlands

**Judith A. C. Rietjens**

‐ Department of Public Health, Erasmus MC, University Medical Center Rotterdam, Rotterdam, The Netherlands
‐ Department of Design, Organization, and Strategy, Faculty of Industrial Design Engineering, Delft University of Technology, Delft, The Netherlands

**Khanh L. N. Luu**

‐ Department of Public Health, Erasmus MC, University Medical Center Rotterdam, Rotterdam, The Netherlands

**Marjolein Lugtenberg**

‐ Department of Dermatology, Erasmus MC, University Medical Center Rotterdam, Rotterdam, The Netherlands
‐ Scientific Center for Care and Welfare (Tranzo), Tilburg School of Social and Behavioral Sciences, Tilburg University, Tilburg, The Netherlands

**Frederika E. Witkamp**

‐ Research Center Innovations in Care, Rotterdam University of Applied Sciences, Rotterdam, The Netherlands
‐ Department of Public Health, Erasmus MC, University Medical Center Rotterdam, Rotterdam, The Netherlands

**Leonieke W. Kranenburg**

‐ Department of Psychiatry, Section of Medical Psychology and Psychotherapy, Erasmus MC, University Medical Center Rotterdam, Rotterdam, The Netherlands

We apologize for this error.

